# CYP2D6-inhibiting drugs and risk of fall injury after newly initiated therapy with beta-blockers—a register-based case-crossover study

**DOI:** 10.1038/s41598-025-09617-4

**Published:** 2025-07-10

**Authors:** Karin Leander, M.-L. Dahl, M. Vikström, J. Möller, K. Söderberg-Löfdal

**Affiliations:** 1 Institute of Environmental Medicine, Unit of Cardiovascular and Nutritional Epidemiology, Karolinska Institutet, Nobels väg 13 Karolinska Institutet, Box 210, 17177 Stockholm, Sweden; 2 Division of Clinical Pharmacology, Department of Laboratory Medicine, Karolinska Institutet, Karolinska University Hospital, Stockholm, Sweden; 3 Department of Global Public Health, Karolinska Institutet, Stockholm, Sweden

**Keywords:** Accidental falls, Beta-blocking agents, Cytochrome P450 CYP2D6 inhibitors, Drug interactions, Health care, Medical research, Risk factors

## Abstract

We assessed whether concomitant use of CYP2D6-inhibiting drugs associates with increased risk of fall injuries after newly initiated therapy with beta-blockers. A case-crossover study design was applied which eliminates confounding from individual fixed characteristics by pairing cases with themselves across time. Data on prescribed and dispensed beta-blockers and hospitalizations for first-time fall injuries in the Swedish population aged ≥ 20 for the period 2006-01-01–2013-12-31 were extracted from national registers. Odds ratios (OR) with 95% confidence interval (CI) of fall injury associated with newly initiated beta-blocker therapy (prescription dispensed within the 28 days preceding the fall injury) while considering concomitant use of CYP2D6-inhibiting drugs, were estimated. Newly initiated beta-blocker therapy (any type) with concomitant use of CYP2D6-inhibiting drugs (any type) was associated with an increased risk of fall injury (OR 1.37, 1.24–1.52). This risk became elevated after restriction to concomitant use of moderate (OR 1.63, 1.26–2.10) or strong CYP2D6 inhibitors (OR 2.25, 1.39–3.63). The results remained similar independent of the beta-blockers’ degree of CYP2D6 metabolism (none, partial or major). Our study indicates the presence of drug-drug interaction for concomitant use of beta-blockers and CYP2D6 inhibitors in relation to the risk of fall injury, irrespective of degree of CYP2D6 metabolism.

**Supplementary Information:**

The online version contains supplementary material available at 10.1038/s41598-025-09617-4.

## Introduction

Fall injuries pose a major threat to health, especially for the elderly. Falls are the second leading cause of accidental or unintentional injury deaths worldwide^1^. A fall accident in an elderly person often has far-reaching negative health consequences related to immobility convalescence. The importance of doctors having a fall-injury prevention perspective when prescribing medications, especially to older adults, has been emphasized^2,3^. Risk factors for fall injuries are many and include increasing age, female sex, environmental factors and factors linked to morbidity and drug therapy^4^ where polypharmacy is one established risk factor^5–8^.

Beta-blockers is one of the most widely used group of medications. They are today mainly used as second-line, add-on treatment of chronic ischemic heart disease, cardiac insufficiency, certain arrhythmia, hypertension and as migraine prophylaxis. Common adverse effects include dizziness, bradycardia, hypotension and orthostatic hypotension, all of which can increase the risk of falls. Studies on the association between beta-blockers and the risk of falls, however, show inconsistent results^9,10^. More studies are needed, and it seems important to consider drug-drug interactions to gain a better understanding of drug-related falls.

The pharmacokinetic properties and elimination pathways of individual beta-blockers vary. For example, metoprolol, the most frequently prescribed beta-blocker in Sweden^11^ is metabolized primarily by the cytochrome P450 (CYP) enzyme CYP2D6^12,13^. Bisoprolol is eliminated to about 50% as metabolites, formed by CYP3A4 and CYP2D6, and to about 50% in unchanged form in urine^14–16^, and the clinical significance of CYP2D6 remains unclear. Alprenolol is eliminated renally without prior metabolism^17^. Inhibition of a particular drug´s major pathway of metabolism potentially increases the plasma exposure of the drug, which in turn may result in increased risks of adverse effects, in particular in the early treatment phase. Indeed, there are pharmacokinetic studies showing that potent inhibitors of CYP2D6 such as paroxetine and fluoxetine may increase the plasma exposure of metoprolol 4- to 6-fold^18–20^, and in some cases, pharmacodynamic effects such as bradycardia and hypotension have been reported^19–21^. Thus, concomitant use of drugs that inhibit CYP2D6 and beta-blockers metabolized to a significant degree by CYP2D6 might increase the risk of falls. Based on this and the fact that side effects of medications often occur at an early stage after the start of treatment^22^, we aimed in this study to determine whether newly initiated treatment with beta-blockers is associated with increased risk of fall injury and whether concomitant use of CYP2D6 inhibiting drugs may have an impact on this association. We considered the adult Swedish population as well as subgroups of age and sex.

## Methods

We conducted a case-crossover study where each fall injury patient also acts as his/her own control^23,24^ based on data from national health registries covering the entire Swedish adult population (about 5 million inhabitants in 2010). The study period was Jan 1st, 2006 – Dec 31st, 2013 and individuals who were 20 years of age or older at index date were considered. We retrieved data from the National Patient Register, which contains information on diagnoses, recorded using international classification of diseases (ICD version 10) codes, during hospital stays for almost all hospital admissions in Sweden since 1987^25^. Fall injuries were identified using the ICD-10 codes W00-W19. Only first-time diagnoses were considered. The date of hospital admission was assumed to be the date of the fall injury. The personal identity number made it possible to link information on hospitalizations to drug dispensations.

Individual-level information on prescribed dispensed drugs, defined using the Anatomical Therapeutic Chemical (ATC) classification codes, was retrieved from the Swedish Prescribed Drug Register (SPDR) which contains data on all dispensed prescribed drugs reported from the pharmacies since July 2005^26^. The SPDR includes data on drug substances, dispensation date and the ATC code. Both beta-blockers (ATC code C07) and the CYP2D6-inhibiting drugs considered in this study are prescription-only in Sweden.

Beta-blockers available for prescription in Sweden during the study period were classified by the authors into one of three categories with respect to CYP2D6 metabolism of potential clinical relevance; those with major CYP2D6 metabolism (metoprolol, timolol, nebivolol and carvedilol), those with partial CYP2D6 metabolism (bisoprolol, propranolol and pindolol) and those metabolized by enzymes other than CYP2D6 or excreted renally (labetalol, sotalol, atenolol). The categorization was mainly based on information in the Flockhart^27^ and the National Medical Information System^28^, with additional information from in vitro metabolic studies, pharmacokinetic studies in individuals pheno- or genotyped for CYP2D6, relevant drug interaction studies, and drug Summary of Product Characteristics (SmPCs).

CYP2D6 inhibitors were identified and classified according to the Cytochrome P450 Drug Interaction Table (“Flockhart table”): Strong (bupropion, cinacalcet, fluoxetine, paroxetine, quinidine), moderate (duloxetine, sertraline, terbinafine), weak (amiodarone, cimetidine) or unspecified (celecoxib, chlorpromazine, citalopram, clemastine, clomipramine, diphenhydramine, doxorubicin, escitalopram, haloperidol, hydroxyzine, levomepromazine, methadone, metoclopramide, moclobemide, perphenazine, ranitidine, ritonavir, ticlopidine)^27^.

**Fig. 1:**
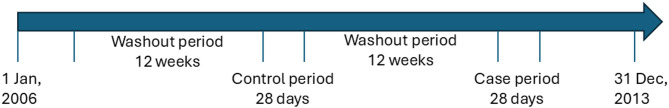
Graphical presentation of the study design, showing the definition of case period, control period and wash-out period for the dispensed prescriptions of beta-blockers. For CYP2D6-inhibiting drugs, prescriptions dispensed during the case period were considered.

The study´s design is outlined in Fig. 1. For a patient to be classified as newly exposed, the beta-blocker prescription had to be dispensed within the 28 days preceding the fall injury and at least one day before the fall accident. To ascertain that the beta-blocker therapy was newly initiated, we used a 12-week washout period prior to the start of the 28-day “case period”, where no dispensations of beta-blocker (any type) should have occurred. The “control period” was set to 112–140 days before the fall injury and beta-blocker exposure during this period was correspondingly assessed. Since any impact on the risk is assumed to occur early after the start of treatment, supplementary analyses were performed with a case period of 14 days.

According to our hypothesis, the drug-drug effect is expected to occur when treatment with beta-blockers is initiated at a time when the person is on medication with a CYP2D6 inhibitor. The CYP2D6 inhibitor does not have to be newly initiated, i.e. no washout period was used. We considered prescriptions of drugs with CYP2D6 inhibitors within the 28-day “case period”.

Analyses were stratified for use of: (1) any CYP2D6–inhibiting drug (strong, moderate, weak or unspecified), and (2) strong or moderate CYP2D6-inhibiting drugs). As very few cases used carvedilol, propranolol or pindolol, only metoprolol was included in the group with major CYP2D6 metabolism, and bisoprolol in the group with partial CYP2D6 metabolism.

To adjust for potential confounding from newly initiated treatment of other potentially fall-inducing drugs, we retrieved data from the SPDR and identified all medications that were newly initiated, correspondingly as for the beta-blockers, and that are known to either cause dizziness or fatigue^28^. For statistical reasons we restricted the selection to medications that occurred in 5% or more of the “case-periods”. This resulted in the selection of two groups of medications for confounding control: anxiolytics (N05B) and hypnotics / sedatives (N05C). Adjustment for potential confounding from antidepressants was not feasible because the majority of antidepressants considered (fluoxetine, paroxetine, sertraline and duloxetine) are moderate or strong CYP2D6 inhibitors.

### Statistical analysis

Analyses were performed with standard methods for matched case-control studies^22^. Each case was considered one stratum and conditional logistic regression was used to estimate odds ratios (OR) with 95% confidence interval (CI) for a first fall injury in relation to a newly initiated beta-blocker therapy. The OR can be considered an estimate of the relative risk^22,29^.

Since underlying causal mechanisms may vary depending on sex, age, and type of fall, we performed analyses stratified for these factors. Age was categorized as 20–59, 60–79 and 80 or above. The type of fall was categorized as “from low level (ICD-10 codes: W00-09, W18)”, “from high level (W10-W17)” and “unspecified (W19)”.

As a sensitivity analysis, we restricted analyses to fall injuries with femur neck (S72.0) or pertrochanteric (S72.1) fractures. This was performed to address the potential risk of reversed causality, as these diagnoses require immediate hospitalization and surgical procedures.

All statistical analyses were performed using the statistical software SAS (version 9.4, SAS institute, Cary, NC, USA).

The project was approved by the Regional Ethical Review Board in Stockholm (reference numbers 2014/856 − 31 and 2015/837 − 32). Since this was a register-based observational study the Swedish Ethical Review Board waived informed consent, as normally done for this type of study. All methods were performed in accordance with the relevant guidelines and regulations.

## Results

### General characteristics

We identified 252,704 fall injury cases, presented in Table 1 by age group, sex, newly initiated beta-blockers and CYP2D6 inhibiting drugs in the 28 days preceding the fall injury. Four out of five cases were found among individuals aged 60 or older. More fall injuries occurred in women (60.7%) than in men. At the time of fall injury, the mean age was 67.2 years for men and 76.1 years for women. In total, 6,863 cases had newly initiated beta-blocker therapy, metoprolol being the most common type. In Supplementary Table 1, the frequency of newly initiated beta-blocker substances, stratified by sex and in 10-year age groups are shown.

**Table 1:** Distribution of the fall injury cases over groups of age, sex, and use of beta-blockers classified according to CYP2D6 metabolism and CYP2D6 inhibiting drugs in the 28-day period before index date*, *N* = 252,704.

Characteristic	Numbers (*N*)	Percentage (%)
Sex		
Male	99,303	39.7
Female	153,401	60.7
Age group (years)		
20–29	9,139	3.6
30–39	8,287	3.3
40–49	13,093	5.2
50–59	21,333	8.4
60–69	34,774	13.8
70–79	49,555	19.6
80–89	84,450	33.4
90+	32,073	13.0
Major CYP2D6 metabolism		
Metoprolol	3,721	1.5
Partial CYP2D6 metabolism		
Bisoprolol	1,003	0.4
No CYP2D6 metabolism		
Labetalol	9	0.004
Sotalol	170	0.07
Atenolol	1,488	0.6
CYP2D6 inhibitors		
All	32,749	12.9
Strong	1632	0.6
Moderate	5059	2.0
Weak	12	0.005
Unspecified	26,857	10.6

*Restricted to cases in which the beta-blocker was newly initiated.

### Risk of fall injury related to newly initiated beta-blocker therapy, irrespectively of degree of CYP2D6 metabolism, by concomitant use of CYP2D6-inhibiting drugs

In the crude analyses, when considering all types of beta-blockers newly initiated during the 28 days period prior to fall injury we observed a small increase in risk without concomitant use of CYP2D6 inhibitors, OR 1.07 (95% CI 1.03–1.11). With concomitant use of a CYP2D6-inhibiting drug (all types considered), the risk was elevated: OR 1.37 (95% CI 1.24–1.52). The risk of fall injury increased further when only moderate CYP2D6 inhibitors were considered and even more when only strong CYP2D6 inhibitors were considered: OR 1.63 (95% CI 1.26–2.10) and OR 2.25 (95% CI 1.39–3.63), respectively.

Corresponding analyses with restriction to beta-blockers initiated during 14 days prior to fall injury showed that beta-blocker therapy without concomitant use of CYP2D6-inhibiting drugs did not affect the risk of fall injury while the risk with a concomitant CYP2D6-inhibiting drug (any type) was increased: OR 1.00 (95% CI 0.95–1.05) versus OR 1.24 (95% CI 1.08–1.41). The increased risk of fall injury associated with the concomitant use of CYP2D6 inhibitors was attenuated when considering moderate CYP2D6 inhibitors only, OR 1.05 (95% CI 0.77–1.45), whereas the risk was elevated when considering strong CYP2D6 inhibitors only, OR 2.73 (95% CI 1.51–4.94).

### Risk of fall injury related to beta-blocker initiation, considering degree of CYP2D6 metabolism, by concomitant use of CYP2D6-inhibiting drugs

Table 2 shows the risks of fall injury after newly initiated beta-blockers (28-day case period) classified in groups according to CYP2D6 metabolism (major, partial and none) and stratified by concomitant use of CYP2D6-inhibiting drugs. The risk estimates without concomitant use of CYP2D6-inhibiting drugs were as follows: major, OR 1.08 (95% CI 1.02–1.13), partial, OR 1.13 (95% CI 1.02–1.24), and no CYP2D6 metabolism, OR 1.03 (95% CI 0.93–1.08). With concomitant use of CYP2D6-inhibiting drugs, risks were increased in all groups: major, OR 1.37 (95% CI 1.19–1.57), partial, OR 1.24 (95% CI 0.95–1.62), and no CYP2D6 metabolism, OR 1.43 (95% CI 1.16–1.76) and even further increased after restriction to strong and moderate CYP2D6 inhibitors (combined category) (Table 2). The results remained similar in separate analyses of men and women (Supplementary Tables 2 and 3). Control for confounding from other fall-inducing drugs did not substantially affect the results, as shown in Supplementary Table 4.

**Table 2:** Risk of fall injury after newly initiated beta-blocker treatment in the case period (1–28 days prior to index date) and the control period (112–140 days prior to index date) stratified by use of CYP2D6-inhibiting drugs 28 days prior to index date.

	No			CYP2D6-inhibiting drugs Yes (all)			Strong and moderate		
	*N*_case_	*N*_control_	OR (95% CI)	*N*_case_	*N*_control_	OR (95% CI)	*N*_case_	*N*_control_	OR (95% CI)
Major CYP2D6 metabolism	3,244	3,014	1.08 (1.02–1.13)	475	347	1.37 (1.19–1.57)	103	73	1.41 (1.05–1.90)
Metoprolol									
Partial CYP2D6 metabolism									
Bisoprolol	884	784	1.13 (1.02–1.24)	119	96	1.24 (0.95–1.62)	35	16	2.18 (1.21–3.95)
No CYP2D6 metabolism	1,453	1,444	1.03 (0.93–1.08)	214	150	1.43 (1.16–1.76)	53	21	2.52 (1.52–4.18)
Labetalol									
Sotalol									
Atenolol									

The beta-blockers are classified according to CYP2D6 metabolism.

N_case,_ Number of subjects with beta-blockers dispensed in the case period and not in the control period; N_control_, Number of subjects with newly initiated beta-blockers dispensed in the control period and not in the case period; OR, odds ratio; CI, confidence interval.

For 60-79-year-olds, there was an increased risk for falls associated with metoprolol without concomitant use of CYP2D6-inhibiting drugs, a risk that became more pronounced with concomitant use of CYP2D6-inhibiting drugs (Table 3). Also, the risks were elevated for bisoprolol and non-CYP2D6 metabolize < d beta-blockers with concomitant use of CYP2D6-inhibiting drugs (Table 3). In the oldest age group (80 years or older), increased risks were found for metoprolol with CYP2D6-inhibiting drugs, for bisoprolol both without CYP2D6-inhibiting drugs and with strong and moderate CYP2D6-inhibiting drugs combined, and for non-CYP2D6 metabolized beta-blockers with strong and moderate CYP2D6-inhibiting drugs combined (Table 4). There were too few exposed cases in the youngest age group (20–59 years of age) to obtain reliable results.

**Table 3:** Risk of fall injury in the age group 60 to 79 years after newly initiated beta-blocker treatment in the case period (1–28 days prior to index date) and the control period (112–140 days prior to index date) stratified by use of CYP2D6-inhibiting drugs 28 days prior to index date.

	No			CYP2D6-inhibiting drugs Yes (all)			Strong and moderate		
	*N*_case_	*N*_control_	OR (95% CI)	*N*_case_	*N*_control_	OR (95% CI)	*N*_case_	*N*_control_	OR (95% CI)
Major CYP2D6 metabolism	1,247	1,088	1.15 (1.06–1.24)	178	122	1.46 (1.16–1.84)	44	33	1.33 (0.85–2.09)
Metoprolol									
Partial CYP2D6 metabolism									
Bisoprolol	312	318	0.98 (0.84–1.15)	55	31	1.77 (1.14–2.76)	16	7	2.29 (0.94–5.56)
No CYP2D6 metabolism	589	578	1.02 (0.91–1.14)	80	50	1.63 (1.14–2.33)	20	12	1.66 (0.81–3.41)
Labetalol									
Sotalol									
Atenolol									

The beta-blockers are classified according to CYP2D6 metabolism.

N_case,_ Number of subjects with beta-blockers dispensed in the case period and not in the control period; N_control_, Number of subjects with newly initiated beta-blockers dispensed in the control period and not in the case period; OR, odds ratio; CI, confidence interval.

**Table 4:** Risk of fall injury in the age group 80 years and older after newly initiated beta-blocker treatment in the case period (1–28 days prior to index date) and the control period (112–140 days prior to index date) stratified by use of CYP2D6-inhibiting drugs 28 days prior to index date.

	No			CYP2D6-inhibiting drugs Yes (all)			Strong and moderate		
	*N*_case_	*N*_control_	OR (95% CI)	*N*_case_	*N*_control_	OR (95% CI)	*N*_case_	*N*_control_	OR (95% CI)
Major CYP2D6 metabolism	1,793	1,739	1.03 (0.97–1.10)	252	205	1.22 (1.02–1.47)	43	35	1.23 (0.79–1.92)
Metoprolol									
PartialCYP2D6 metabolism									
Bisoprolol	532	426	1.25 (1.10–1.42)	58	62	0.94 (0.65–1.34)	17	8	2.12 (0.92–4.92)
No CYP2D6 metabolism	770	784	0.98 (0.89–1.08)	115	95	1.21 (0.92–1.59)	27	8	3.38 (1.53–7.43)
Labetalol									
Sotalol									
Atenolol									

The beta-blockers are classified according to CYP2D6 metabolism.

N_case_, Number of subjects with beta-blockers dispensed in the case period and not in the control period; N_control_, Number of subjects with newly initiated beta-blockers dispensed in the control period and not in the case period; OR, odds ratio; CI, confidence interval.

After restriction to femur and pertrochanteric fractures as well as stratification by fall from high, low or unspecified levels, results were not substantially different from the main results (Table S5, Table S6).

## Discussion

Our study showed that the use of beta-blockers without concomitant use of CYP2D6-inhibiting drugs was associated with a slightly elevated risk of fall injury. The risk was further elevated with concomitant use of CYP2D6-inhibiting drugs, and with increasing potency of inhibition. However, when we considered the degree of CYP2D6 metabolism of beta-blockers, we found no clear effects on the outcome and therefore our study does not provide strong support for our hypothesis that CYP2D6 inhibition is of importance for the effects of beta-blockers on the risk of fall injuries.

All three sub-groups of beta-blockers considered (major [metoprolol], partial [bisoprolol] and no CYP2D6 metabolism) showed slightly elevated risk for fall injury without concomitant use of CYP2D6-inhibiting drugs and further increased risks with concomitant CYP2D6-inhibiting drug use, especially when restricted to strong/moderate inhibitors (combined category). The results concerning beta-blockers with major CYP2D6 metabolism are in line with our hypothesis that inhibition of a major metabolic pathway results in elevated drug concentrations in the blood which may increase the risk of falls. The quantitative impact of CYP2D6 in the metabolism of bisoprolol is unclear. However, as the risk estimates during concomitant use of CYP2D6 inhibitors were also increased for beta-blockers with no CYP2D6 metabolism, it is plausible that polypharmacy as such, or, more specifically the use of the drugs classified as moderate/strong CYP2D6 inhibitors influenced the risks. Therefore, our results are not conclusive concerning the role of pharmacokinetic interactions as a factor influencing fall risk.

Our study further showed that the risk of falls associated with metoprolol was pronounced in the presence of concomitant use of CYP2D6-inhibiting drugs in 60-79-year-olds, but not in the oldest age group, implying that other factors apart from CYP2D6 inhibition may be of importance in older persons. Possibly, increased sensitivity to pharmacodynamic effects and interactions is more important than pharmacokinetic interactions in the oldest part of the population.

As far as we know, there is no previous study of the initiation of beta-blocker therapy in relation to risk of fall injury investigating potential impact from concomitant drug use. This limits comparison of results with previous literature. However, our group previously investigated other fall inducing drugs (opioids, antidepressants and antipsychotics) and concomitant use of CYP2D6 inhibiting drugs, using the same methodology, and found indications of clinically relevant drug-drug interactions^24,30^.

### Study strengths and limitations

An important strength of the present study is the use of a case-crossover design, as it has advantages for studying acute drug-related effects on health^29^. In particular, the case-crossover design has the important advantage that between-subject differences in measured or unmeasured time-invariant confounders, such as chronic diseases and lifestyle, are avoided.

Another major strength is the use of national registers with high coverage of the population for hospitalizations; 99% of hospital discharge primary diagnoses are covered^25^. In a validation study using ambulance records as the golden standard, the agreement for fall-related injuries (W00-W19) in the National Patient Register was found to be 93.9%^31^. Of note, the fall-related injury is diagnosed by a hospital physician independent of dispensed prescribed drugs at the pharmacies.

The validity of our results is also supported by the consistent findings from our sensitivity analyses where restriction was made to femur neck and femur pertrochanteric fractures.

The limitations of our study include a potential misclassification of beta-blocker use and use of CYP2D6-inhibiting drugs due to poor compliance. No information on actual intake was available. However, considering that compliance is generally highest in the initiation phase^32^, this would not have any substantial impact on our results. Further, any misclassification would likely bias our results towards the null. Another source of misclassification concerns treatment with CYP2D6-inhibiting drugs during the case period; we have assumed that drug dispensations during the case period imply exposure throughout the case period, but it is both possible that treatment had been completed by the time the beta-blocker therapy was initiated and possible that there was continued treatment based on a prescription that occurred before the case period. This misclassification would also likely bias our results towards the null. Since some herbal preparations, such as St. John’s wort, have similar effects as CYP2D6 inhibitors^33^, CYP2D6 inhibitory effects could be underestimated to some extent in our study. Such bias would likely also bias our results towards the null.

Another limitation is related to that the SPDR does not include information on drugs used in hospitals. However, beta-blockers are predominantly prescribed in outpatient settings.

Finally, although the chosen study design effectively eliminates confounding, uncontrolled confounding by time varying factors cannot be excluded.

### Clinical implications

Our results provide some support for the hypothesis of an interaction between beta-blockers and CYP2D6-inhibiting drugs but not fully because no clear difference was seen when stratifying for the degree of CYP2D6 metabolism of beta-blockers. However, with stronger CYP2D6 inhibitors, the association with fall risk became stronger, indicating that CYP2D6 inhibitors are important for how newly started beta-blockers affect the risk of fall injury. Current guidelines for falls prevention emphasize that older adults at high risk of falling should be assessed for fall-risk increasing drugs (FRIDs) and that medication review including modification or withdrawal of FRIDs should be considered^34,35^. Of the FRIDs, antihypertensive drugs, including beta-blockers, make up a significant proportion and have repeatedly been shown to be associated with an increased risk of falls, especially in the elderly^36,37^. However, a systematic review concluded paucity of evidence for that reduced use of FRIDs would prevent fall injuries in the older^38^. Although there is no clear evidence that the initiation of beta-blockers poses a risk of falls^39^, our results suggest the need for vigilance in the initiation phase of this group of drugs. In this context, it is worth considering that the use of antihypertensive drugs is widespread and increasing; in Sweden, 21% of men and women filled a prescription for antihypertensives in 2017, and during 2017–2021 the prescription rate increased by 5% among men and 3% among women^11^. Even small increases in risk have major consequences because the number of individuals who initiates treatment with these drugs is so large.

## Conclusions

Newly initiated treatment with beta-blockers was associated with a slightly increased short-term risk of fall injury in adults 20 years or older. The results are based primarily on the age group 60 years or older which had the highest number of exposed. Concomitant use of CYP2D6-inhibiting drugs strengthened this association, irrespective of the degree of CYP2D6 metabolism of individual beta-blockers. Thus, our study points to the existence of clinically relevant drug-drug interactions, which are possibly more of pharmacodynamic than of pharmacokinetic character among older adults.

## Electronic supplementary material

Below is the link to the electronic supplementary material.

Supplementary Material 1

## Data Availability

The datasets analyzed during the current study are retrieved from the Swedish National Patient Registry and the Swedish Prescribed Drug Registry. They are not publicly available due to ethics permits restricting redistribution of data but are available from the corresponding author upon reasonable request.
