# Supplementary material for: CYP2D6-inhibiting drugs and risk of fall injury after newly initiated therapy with beta-blockers—a register-based case-crossover study
Source: Sci Rep. 2025 Jul 10;15:24918. doi: 10.1038/s41598-025-09617-4 (PMC12246248; doi:10.1038/s41598-025-09617-4)
Supplement: Supplementary file 1 — Supplementary Material 1 [file 41598_2025_9617_MOESM1_ESM.pdf]

SUPPLEMENTARY MATERIAL

**Table S1.** Frequency of newly initiated beta-blocker substances, stratified by sex and age group

| | Men | Women | 20-29 years | | 30-39 years | | 40-49 years | | 50-59 years | | 60-69 years | | 70-79 years | | 80-89 years | |
|---|---|---|---|---|---|---|---|---|---|---|---|---|---|---|---|---|
| | (total) | (total) | Men | Women | Men | Women | Men | Women | Men | Women | Men | Women | Men | Women | Men | Women |
| **Major CYP2D6 metabolism** | | | | | | | | | | | | | | | | |
| Metoprolol, C07AB02 | 1426 | 2245 | 2 | 2 | 6 | 5 | 36 | 19 | 82 | 93 | 260 | 221 | 391 | 529 | 541 | 1036 |
| Metoprolol (and felodipine), C07FB02 | 16 | 34 | 1 | 3 | 4 | 9 | 7 | 4 | 3 | 10 | 1 | 8 | 0 | 0 | 0 | 0 |
| Carvedilol, C07AG02 | 45 | 70 | 3 | 1 | 11 | 1 | 10 | 3 | 19 | 9 | 2 | 15 | 0 | 34 | 0 | 7 |
| *Total* | 1487 | 2349 | 6 | 6 | 21 | 15 | 53 | 26 | 104 | 112 | 263 | 244 | 391 | 563 | 541 | 1043 |
| **Partial CYP2D6 metabolism** | | | | | | | | | | | | | | | | |
| Bisoprolol, C07AB07 | 402 | 600 | 3 | 3 | 3 | 18 | 19 | 59 | 56 | 137 | 114 | 305 | 163 | 78 | 44 | 0 |
| Propranolol, C07AA05 | 94 | 222 | 4 | 2 | 3 | 10 | 6 | 21 | 26 | 38 | 11 | 49 | 25 | 78 | 16 | 34 |
| Pindolol, C07AA03 | 15 | 34 | 2 | 1 | 2 | 9 | 3 | 16 | 7 | 8 | 1 | 0 | 0 | 0 | 0 | 0 |
| *Total* | 511 | 856 | 9 | 6 | 8 | 37 | 28 | 96 | 89 | 183 | 126 | 354 | 188 | 156 | 60 | 34 |
| **No CYP2D6 metabolism** | | | | | | | | | | | | | | | | |
| Labetalol, C07AG01 | 7 | 2 | 1 | 1 | 6 | 1 | 0 | 0 | 0 | 0 | 0 | 0 | 0 | 0 | 0 | 0 |
| Sotalol, C07AA07 | 65 | 105 | 2 | 5 | 5 | 31 | 14 | 55 | 38 | 14 | 6 | 0 | 0 | 0 | 0 | 0 |
| Atenolol , C07AB03 | 456 | 1032 | 1 | 2 | 9 | 10 | 40 | 47 | 91 | 104 | 142 | 271 | 137 | 451 | 36 | 147 |
| *Total* | 528 | 1139 | 4 | 8 | 20 | 42 | 54 | 102 | 129 | 118 | 148 | 271 | 137 | 451 | 36 | 147 |

**Table S2**. Risk of fall injury in men after newly initiated beta-blocker therapy in the case period (1-28 days prior to index date) and the control period (112-140 days prior to index date) stratified by use of CYP2D6-inhibiting drugs 28 days prior to index date. The beta-blockers are classified according to CYP2D6 metabolism.

| | CYP2D6-inhibiting drugs | | | | | | | | |
|---|---|---|---|---|---|---|---|---|---|
| | No | | | Yes (all) | | | Strong and moderate | | |
| | Ncase | Ncontrol | OR (95% CI) | Ncase | Ncontrol | OR (95% CI) | Ncase | Ncontrol | OR (95% CI) |
| Major CYP2D6 metabolism | | | | | | | | | |
| Metoprolol | 1290 | 1145 | 1.13 (1.04- 1.22) | 152 | 125 | 1.22 (0.96-1.54) | 32 | 27 | 1.19 (0.71-1.98) |
| Partial CYP2D6 metabolism | | | | | | | | | |
| Bisoprolol | 357 | 318 | 1.12 (0.97- 1.31) | 45 | 29 | 1.55 (0.97-2.48) | 14 | 3 | 4.67 (1.34-16.24) |
| No CYP2D6 metabolism (Labetalol, Sotalol, Atenolol) | 472 | 460 | 1.03 (0.90- 1.17) | 56 | 34 | 1.65 (1.08-2.52) | 10 | 5 | 2.00 (0.68-5.85) |

$N_{case}$, Number of subjects with beta-blockers dispensed in the case period and not in the control period; $N_{control}$, Number of subjects with newly initiated beta-blockers dispensed in the control period and not in the case period; OR, odds ratio; CI, confidence interval

**Table S3.** Risk of fall injury in women after newly initiated beta-blocker therapy in the case period (1-28 days prior to index date) and the control period (112-140 days prior to index date) stratified by use of CYP2D6-inhibiting drugs 28 days prior to index date. The beta-blockers are classified according to CYP2D6 metabolism.

| | CYP2D6-inhibiting drugs | | | | | | | | |
|---|---|---|---|---|---|---|---|---|---|
| | No | | | Yes (all) | | | Strong and moderate | | |
| | Ncase | Ncontrol | OR (95% CI) | Ncase | Ncontrol | OR (95% CI) | Ncase | Ncontrol | OR (95% CI) |
| Major CYP2D6 metabolism | | | | | | | | | |
| Metoprolol | 1,954 | 1,869 | 1.05 (0.98-1.11) | 323 | 222 | 1.45 (1.23-1.72) | 71 | 46 | 1.54 (1.07-2.24) |
| Partial CYP2D6 metabolism | | | | | | | | | |
| Bisoprolol | 527 | 466 | 1.13 (0.99-1.28) | 74 | 67 | 1.10 (0.79-1.54) | 21 | 13 | 1.62 (0.81-3.23) |
| No CYP2D6 metabolism (Labetalol, Sotalol, Atenolol) | 981 | 984 | 1.00 (0.91-1.09) | 158 | 116 | 1.36 (1.07-1.73) | 43 | 16 | 2.69 (1.51-4.77) |

$N_{case}$, Number of subjects with beta-blockers dispensed in the case period and not in the control period; $N_{control}$, Number of subjects with newly initiated beta-blockers dispensed in the control period and not in the case period; OR, odds ratio; CI, confidence interval

**Table S4.** Risk, after single adjustments for potential confounders, of fall injury after newly initiated beta-blocker therapy in the case period (1-28 days prior to index date) and the control period (112-140 days prior to index date) stratified by use of CYP2D6-inhibiting drugs 28 days prior to index date. The beta-blockers are classified according to CYP2D6 metabolism.

| | CYP2D6-inhibiting drugs | | | | | | | | |
|---|---|---|---|---|---|---|---|---|---|
| | No | | | Yes (all) | | | Strong and moderate | | |
| | Ncase | Ncontrol | OR (95% CI) | Ncase | Ncontrol | OR (95% CI) | Ncase | Ncontrol | OR (95% CI) |
| Major CYP2D6 | | | | | | | | | |
| Metoprolol | 3244 | 3014 | 1.08 (1.02-1.13) | 475 | 347 | 1.37 (1.19-1.57) | 103 | 73 | 1.41 (1.05-1.90) |
| Adjusted[1] | | | 1.08 (1.03-1.13) | | | 1.33 (1.15-1.53) | | | 1.33 (1.01-1.86) |
| Adjusted[2] | | | 1.07 (1.02-1.13) | | | 1.26 (1.09-1.46) | | | 1.27 (0.93-1.74) |
| Minor CYP2D6 | | | | | | | | | |
| Bisoprolol | 884 | 784 | 1.13 (1.02-1.24) | 119 | 96 | 1.24 (0.95-1.62) | 35 | 16 | 2.18 (1.21-3.95) |
| Adjusted[1] | | | 1.14 (1.03-1.25) | | | 1.22 (0.93-1.61) | | | 2.19 (1.21-3.98) |
| Adjusted[2] | | | 1.13 (1.02-1.24) | | | 1.16 (0.88-1.52) | | | 2.37 (1.24-4.51) |
| No CYP2D6 metabolism | 1453 | 1444 | 1.03 (0.93-1.08) | 214 | 150 | 1.43 (1.16-1.76) | 53 | 21 | 2.52 (1.52-4.18) |
| Adjusted[1] | | | 1.01 (0.94-1.09) | | | 1.35 (1.08-1.68) | | | 2.29 (1.36-3.85) |
| Adjusted[2] | | | 1.00 (0.93-1.08) | | | 1.25 (1.00-1.57) | | | 2.44 (1.32-4.49) |

$N_{case}$, Number of subjects with beta-blockers dispensed in the case period and not in the control period; $N_{control}$, Number of subjects with newly initiated beta-blockers dispensed in the control period and not in the case period; OR, odds ratio; CI, confidence interval

[1]Adjusted for anxiolytics (N05B)

[2]Adjusted for hypnotics / sedatives (N05C)

**Table S5.** Risk of fall injury, restricted to femur (S72.00-S72.01) and pertrochanteric (S72.10-S72.11) fracture, after newly initiated beta-blocker therapy in the case period (1-28 days prior to index date) and the control period (112-140 days prior to index date) stratified by use of CYP2D6-inhibiting drugs 28 days prior to index date. The beta-blockers are classified according to CYP2D6 metabolism.

| | CYP2D6-inhibiting drugs | | | | | | | | |
|---|---|---|---|---|---|---|---|---|---|
| | No | | | Yes (all) | | | Strong and moderate | | |
| | Ncase | Ncontrol | OR (95% CI) | Ncase | Ncontrol | OR (95% CI) | Ncase | Ncontrol | OR (95% CI) |
| Major CYP2D6 metabolism | | | | | | | | | |
| Metoprolol | 797 | 777 | 1.03 (0.93-1.13) | 139 | 108 | 1.29 (1.00-1.65) | 27 | 21 | 1.29 (0.73-2.27) |
| Minor CYP2D6 metabolism | | | | | | | | | |
| Bisoprolol | 207 | 198 | 1.05 (0.86-1.27) | 35 | 36 | 0.97 (0.61-1.55) | 10 | 4 | 2.50 (0.78-7.97) |
| No CYP2D6 metabolism (Labetalol, Sotalol, Atenolol) | 385 | 398 | 0.97 (0.84-1.11) | 64 | 51 | 1.25 (0.87-1.81) | 7 | 7 | 1.00 (0.36-2.85) |

$N_{case}$, Number of subjects with beta-blockers dispensed in the case period and not in the control period; $N_{control}$, Number of subjects with newly initiated beta-blockers dispensed in the control period and not in the case period; OR, odds ratio; CI, confidence interval

**Table S6**. Risk of fall injury, stratified by fall from high, low or unspecified height after newly initiated beta-blocker therapy in the case period (1-28 days prior to index date) and the control period (112-140 days prior to index date) stratified by use of CYP2D6-inhibiting drugs 28 days prior to index date. The beta-blockers are classified according to CYP2D6 metabolism.

| | CYP2D6-inhibiting drugs | | | | | | | | |
|---|---|---|---|---|---|---|---|---|---|
| | No | | | Yes (all) | | | Strong and moderate | | |
| Major CYP2D6 metabolism | Ncase | Ncontrol | OR (95% CI) | Ncase | Ncontrol | OR (95% CI) | Ncase | Ncontrol | OR (95% CI) |
| High | 281 | 263 | 1.07 (0.90-1.26) | 28 | 15 | 1.87 (1.00-3.49) | 9 | 1 | 9.00 (1.14-71.03) |
| Low | 2215 | 2035 | 1.09 (1.02-1.16) | 323 | 250 | 1.29 (1.10-1.52) | 68 | 53 | 1.28 (0.90-1.84) |
| Unspecified | 748 | 716 | 1.04 (0.94-1.16) | 124 | 82 | 1.51 (1.14-2.00) | 26 | 19 | 1.37 (0.75-2.47) |
| | | | | | | | | | |
| Minor CYP2D6 metabolism | | | | | | | | | |
| High | 63 | 54 | 1.17 (0.81-1.68) | 6 | 3 | 2.00 (0.50-8.00 | 3 | 0 | n/a |
| Low | 604 | 535 | 1.13 (1.00-1.27) | 83 | 74 | 1.14 (0.83-1.56) | 22 | 12 | 1.83 (0.91-3.70) |
| Unspecified | 217 | 95 | 1.11 (0.92-1.35) | 30 | 20 | 1.50 (0.85-2.64) | 10 | 4 | 2.50 (0.78-7.97) |
| | | | | | | | | | |
| No CYP2D6 metabolism | | | | | | | | | |
| High | 117 | 146 | 0.80 (0.63-1.02) | 19 | 6 | 3.17 (1.26-7.92) | 5 | 1 | 5.00 (0.58-42.79) |
| Low | 1011 | 968 | 1.04 (0.96-1.14) | 146 | 113 | 1.29 (1.01-1.65) | 38 | 13 | 2.92 (1.56-5.49) |
| Unspecified | 325 | 330 | 0.98 (0.84-1.15) | 49 | 31 | 1.58 (1.01-2.48) | 10 | 7 | 1.43 (0.54-3.75) |

$N_{case}$, Number of subjects with beta-blockers dispensed in the case period and not in the control period; $N_{control}$, Number of subjects with newly initiated beta-blockers dispensed in the control period and not in the case period; OR, odds ratio; CI, confidence interval
